# Dementia Diagnosis & Outmigration of Older Puerto Rican Medicare Fee-for-Service Beneficiaries, 2012-2019 Trends

**DOI:** 10.1093/geroni/igaf122.1504

**Published:** 2025-12-31

**Authors:** Jeung Hyun Kim, Yoojin Lee, Yanru Liao, Monica Colon-Vargas, Maricruz Rivera-Hernandez

**Affiliations:** Brown University, Brown University, Rhode Island, United States; Brown University, Providence, Rhode Island, United States; Brown University Center for Gerontology and Healthcare Research, Providence, Rhode Island, United States; Brown University, Providence, Rhode Island, United States; Brown University School of Public Health, Providence, Rhode Island, United States

## Abstract

Prior research shows that older Puerto Ricans may be migrating to the US mainland, which may include those with an Alzheimer’s Disease and Related Dementia (ADRD) diagnosis (Rivera-Hernandez, 2022; Kim et al., 2024). The purpose of this research was to examine yearly out-migration trends among older Puerto Ricans by examining their movement patterns from 2012-2019, which included the period of Hurricane Maria. The analysis used data from the Master Beneficiary Summary File and the American Community Survey, and focused on Medicare fee-for-service enrollees in Puerto Rico (65+). The Chronic Conditions Warehouse algorithm was used to ascertain ADRD diagnosis, and migration was defined as the change of (end-of-year) residence from one year to the next. A linear probability model was used to calculate differences in the rate of outmigration between beneficiaries with and without ADRD. There were about 44,000∼66,000 beneficiaries each year and around 15∼16% with ADRD. Outcomes showed that, overall, older Puerto Ricans previously diagnosed with ADRD were slightly more likely to move to the US mainland compared to those without an ADRD diagnosis across the years (Adjusted differences ranged, b = 0.0014∼0.0041), with the highest proportion migrating during the year of Hurricane Maria (over 2.5% in 2017). However, the difference was not statistically significant for some years. In sensitivity analyses across groups with sociodemographic and clinical characteristics, the likelihood of outmigration was higher among those with ADRD compared to those without ADRD. Our results suggest that older adults with complex health needs appear to be out-migrating following their ADRD diagnosis.

